# The relation between the patient health questionnaire-15 and DSM somatic diagnoses

**DOI:** 10.1186/s12888-016-1068-2

**Published:** 2016-10-18

**Authors:** Shih-Cheng Liao, Wei-Lieh Huang, Huei-Mei Ma, Min-Tzu Lee, Tzu-Ting Chen, I-Ming Chen, Susan Shur-Fen Gau

**Affiliations:** 1 Department of Psychiatry, National Taiwan University Hospital, No.7, Zhongshan S. Rd, Zhongzheng Dist Taipei City, 100 Taiwan (Republic of China); 2 Department of Psychiatry, College of Medicine, National Taiwan University, No.1, Sec. 1, Ren’ai Rd, Zhongzheng Dist Taipei City, 100 Taiwan (Republic of China); 3 Department of Psychiatry, National Taiwan University Hospital, Yun-Lin Branch, No.579, Sec. 2, Yunlin Rd, Douliu City, Yunlin County 64041 Taiwan (Republic of China); 4 Graduate Institute of Clinical Medicine, National Taiwan University, No.7, Zhongshan S. Rd, Zhongzheng Dist Taipei City, 100 Taiwan (Republic of China); 5 Institute of Health Policy and Management, College of Public Health, National Taiwan University, Rm. 651, 6 F., No.17, Xuzhou Rd, Zhongzheng Dist Taipei City, 100 Taiwan (Republic of China)

**Keywords:** Patient Health Questionnaire-15, Somatoform disorders, Somatic symptom and related disorders, Somatic symptom disorder

## Abstract

**Background:**

Our purpose was to examine the reliability and validity of the Chinese version of the Patient Health Questionnaire-15 (PHQ-15) in Taiwan, and to explore its relation to somatoform disorders (DSM-IV) and to somatic symptom and related disorders (DSM-5).

**Methods:**

We recruited 471 individuals, 151 with somatoform disorders and 200 with somatic symptom and related disorders. Subjects completed the Chinese version of the PHQ-15, Beck Depression Inventory-II (BDI-II), Beck Anxiety Inventory (BAI), and received a DSM-IV- and DSM-5-based diagnostic interview. We performed exploratory factor analysis and assessed test-retest reliability, internal consistency, and correlation with BDI-II/BAI to confirm reliability and validity, and carried out ROC curve analysis to determine suitability for evaluation or screening purposes. PHQ-15 scores were compared between patients with various DSM-IV psychiatric diagnoses (such as DSM-IV somatoform disorders, panic disorder, other anxiety/depressive disorders) or no DSM-IV diagnosis and patients with DSM-5 somatic symptom and related disorders or no DSM-5 diagnosis.

**Results:**

The Chinese version identified cardiopulmonary, pain-fatigue, and gastrointestinal as major factors and had good reliability (0.803–0.930), internal consistency (0.637–0.861), and correlation coefficients with BDI-II/BAI (0.407–0.619, 0.536–0.721, respectively). The PHQ-15 scores were similar in patients with somatoform disorders and patients with panic disorder; higher in patients with somatoform disorders and panic disorder than in patients with other anxiety/depressive disorders; and significantly higher in patients with somatic symptom and related disorders than in patients without this diagnosis. The AUC of the PHQ-15 was 0.678 (cutoff 6/7) for screening somatoform disorders (DSM-IV) and 0.725 (cutoff 4/5) for screening somatic symptom and related disorders (DSM-5).

**Conclusions:**

The Chinese version of the PHQ-15 is suitable for evaluating somatic symptom and related disorders. The preponderance of somatic symptom disorder in our sample, lack of evaluation of functional disorders, and recruitment solely from psychiatric clinics are possible limitations.

**Electronic supplementary material:**

The online version of this article (doi:10.1186/s12888-016-1068-2) contains supplementary material, which is available to authorized users.

## Highlights

1. The Chinese version of PHQ-15 focuses on 3 major factors.
2. Its validity and reliability are satisfactory.
3. The AUC of the PHQ-15 is higher for screening somatic symptom and related disorders than for screening somatoform disorders.

## Background

Psychiatric disorders presenting with mainly somatic symptoms are described in the Diagnostic and Statistical Manual of Mental Disorders (DSM) and International Classification of Diseases (ICD). Although the gold standard method for diagnosing a DSM or ICD psychiatric disorder is the diagnostic interview, it is hard to interview all subjects in large-scale epidemiological studies. Therefore, it is clinically important to determine whether self-administered psychometric tools plus diagnostic criteria can be used for screening or evaluating the severity of this group of disorders. Some of these tools include the World Health Organization Schedule for Somatoform Disorders Screener, the Symptom Checklist-12, and the widely used Patient Health Questionnaire-15 (PHQ-15) [1]. Developed by Kroenke et al., the PHQ-15 is the self-administered version of the Primary Care Evaluation of Mental Disorders (PRIME-MD) [1, 2]. Differing from the depression-oriented Patient Health Questionnaire-9 (PHQ-9), the PHQ-15 is focused on somatic distress. It contains 15 items rated on a 3-point Likert scale (ranging from 0 [not bothered at all] to 2 [bothered a lot]) [1]. Many studies have shown that the PHQ-15 has good content validity and reliability [3–6], and is suitable for screening or evaluating somatoform disorders defined by the DSM-IV [7–9].

One of the changes from the DSM-IV to the DSM-5 is in the concept of somatic symptoms. The “somatoform disorders” have become “somatic symptom and related disorders”. Whereas the emphasis of the DSM-IV is on “medically unexplained,” that of the DSM-5 is on the “distress” of somatic discomforts [10]. Because the term “medically unexplained” reflects the absence of clear diagnosis, this change of emphasis in the DSM-5 may increase not only diagnostic consistency, but also the size of this group of disorders. Though developed in the DSM-IV era, the 15 items of the PHQ-15 do not specifically ask about “medically unexplained” symptoms [1]. Therefore, it may also be used to evaluate somatic symptom and related disorders in the DSM-5. Evidence shows the applicability of the PHQ-15 to evaluation of somatic symptom disorder as defined by the DSM-5 [11]. In a systematic review, the PHQ-15 was the most psychometrically valid and useful of all somatic symptom questionnaires [12]. Moreover, the PHQ-15 has also been used in studies of functional disorders (such as fibromyalgia, irritable bowel syndrome, chronic pelvic pain) [13–19], other psychiatric disorders (such as depressive disorder and anxiety disorder) [20–23], and physical diseases (such as benign prostate hypertrophy) [24, 25].

The PHQ-15 has been translated into many languages. The Korean version (which is reported to have good reliability and content validity [4] was used to explore the relation between somatic and depressive symptoms [26]. The Chinese version had satisfactory internal consistency and test-retest reliability in a Hong Kong population [27]. High score on the PHQ-15 was shown to be associated with female gender, young age, low educational level, and low economic status [27]. Studies in China show that somatization (as defined by the PHQ-15) is highly associated with depression and anxiety [28] and that a cutoff of 10 points is suitable for the detection of somatizers [29]. Even though the PHQ-15 has yet to be applied in Taiwan, we consider it a suitable tool for evaluating somatic symptoms in Taiwan. Because the description of somatic symptoms varies between China, Hong Kong, and Taiwan, a Chinese version of the PHQ-15 is needed specifically for the population in Taiwan.

For exploring the above issues, we translated the PHQ-15 into Taiwanese Mandarin (Traditional Chinese Mandarin that includes Taiwanese vocabulary) and applied it to our investigation of somatic symptoms in Taiwan. The 3 aims of our present study were to 1) determine the content reliability and validity of the Chinese version of the PHQ-15; 2) clarify the relationships among different types of somatic symptoms in the Taiwanese population; and 3) investigate whether the PHQ-15 is suitable for screening purposes and for evaluating the severity of somatoform disorders (DSM-IV) and somatic symptom and related disorders (DSM-5).

## Methods

### Participants and procedure

This study was performed after getting the approval of Institutional Review Board of National Taiwan University Hospital. We recruited subjects from 2 sites: National Taiwan University Hospital (which is located in an urban region of Taiwan) and National Taiwan University Hospital, Yun-Lin Branch (which is in a rural area of Taiwan). Individuals were recruited from the psychiatric outpatient clinics and communities near the 2 hospitals. Our study targets were: (1) patients with depression, anxiety, or somatic symptoms followed in psychiatric clinics; (2) healthy subjects without physical or psychiatric illnesses. Exclusion criteria were: (1) having psychotic symptoms or reality disturbance; (2) having cognitive impairment or difficulty reading questionnaires; (3) age lower than 15 or higher than 70 years old (parents of subjects lower than 20 years old completed parental consents); (4) having a life-threatening physical disease. After completing the informed consent form, the individuals received a diagnostic interview and completed the Chinese version of the PHQ-15, Beck Depression Inventory-II (BDI-II), and Beck Anxiety Inventory (BAI). Other demographic factors were recorded by research assistants. To assess test-retest reliability, some subjects completed the PHQ-15 again 2 weeks later.

### Construction of psychiatric diagnoses

Clinical interview was performed by 4 board-certified psychiatrists. The interview was conducted in 3 parts. The first part focused on somatoform disorders of the DSM-IV-TR and used the Structured Clinical Interview for DSM-IV Axis I Disorders (SCID-1), Module G, to ensure consistency. The second part examined all DSM-IV-TR diagnoses other than somatoform disorders. The third part was solely about somatic symptom and related disorders in the DSM-5. Because the structured interview does not cover DSM-5 diagnoses, this part was based on the criteria for somatic symptom disorder (SSD) including the existence of 1 or more distressing somatic symptoms (SSD criterion A); having cognitive, emotional or behavioral features such as catastrophizing cognitive style, persistent high level of health anxiety, or abnormal illness behavior related to somatic symptoms (SSD criterion B); and presence of the above symptoms for at least 6 months (SSD criterion C).

### The translation of the Chinese version of the patient health questionnaire-15

The authors of the original version of the PHQ-15 were contacted and agreed to this translation. The translation was performed in 3 steps. Firstly, a psychiatric specialist translated the English version of the PHQ-15 into traditional Chinese but using a vocabulary common in Taiwan. Secondly, 2 specialists in psychosomatics checked and modified the Chinese version. Finally, a backward translation was performed by another specialist to confirm consistency.

### Other psychometric measurements

The Beck Depression Inventory-II (BDI-II), a 21-question self-administered questionnaire, developed by Aaron T. Beck in 1996 [30], with items evaluated on a 4-point Likert scale (0 for not at all to 3 for severely) can be used for individuals older than 13 years. Individuals are asked to rate their mood status 2 weeks before the assessment. The BDI-II has been proven to have good content validity and reliability and excellent test-retest reliability and internal consistency (i.e., 0.93 and 0.91, respectively) [30]. Summated scores of 14–19, 20–28, and 29–63 indicate mild, moderate, and severe depression, respectively. The content validity and reliability of the Chinese version of the BDI-II (used in our study) was previously assessed by [31]. Internal consistency of BDI-II in our sample was 0.94.

The Beck Anxiety Inventory (BAI) [32] is similar to the BDI-II; is a self-administered questionnaire consisting of 21 4-point Likert scale items (rated 0 for not at all to 3 for severely); is suitable for individuals aged 17–80 years, and emphasizes the somatic aspects of anxiety (panic-like). Individuals answer the questions based on their current feelings. It has good test-retest reliability of 0.75, good internal consistency of 0.85, and moderate correlation with BDI-II, STAI-state, and STAI-trait (0.66, 0.47, 0.58, respectively) [30, 33]. The cutoffs of the BAI are 8, 16, and 26 points. The Chinese version of the BAI used in our study also had good content reliability and validity [34]. Its internal consistency in our sample was 0.95.

### Statistical analysis

SPSS (version 19) and LISREL (version 8.51) were used for statistical analysis. We used the Shapiro-Wilk test on all continuous variable data at first. If the result was not compatible with a normal distribution, a non-parametric method would be adopted in further steps. Using the PHQ-15 data of all subjects, exploratory factor analysis (with principal component analysis for extraction and varimax for factor rotation) was performed to clarify the relationship among items. A specific latent structure described by Kroenke et al. [2] was then examined with confirmatory factor analysis. The test-retest reliability (using the intraclass correlation coefficient) and internal consistency (using Cronbach’s alpha) of PHQ-15 total scores and 3 major factors identified in exploratory factor analysis were assessed. The associations of PHQ-15 total scores, PHQ-15 major factor scores, BDI-II score, and BAI score were evaluated using Pearson’s correlation analysis.

The results of the diagnostic interview were used to analyze the relation between the PHQ-15 and clinical diagnoses. Based on the DSM-IV-TR, subjects were divided into a panic disorder group, somatoform disorders group, other neurotic disorders group, and others without psychiatric diagnoses group. ANOVA (with post-hoc analysis using the Scheffé method) was performed to compare the PHQ-15 scores of the 4 groups. ROC curve analysis was used to determine the optimal cutoff value of the PHQ-15 score for somatoform disorders. Based on the DSM-5, subjects were divided into groups with/without somatic symptom and related disorders, and an independent *t* test was used to compare PHQ-15 scores between these groups. Finally, cutoffs for different severities (mild, moderate, and severe) of SSD (the most common diagnosis in our subjects with somatic symptom and related disorders) were determined from the ROC curve. An ROC curve was used because SSD can be defined in terms of severity, and different severities can be viewed as categorical variables.

## Results

### Demographic data and psychiatric diagnoses of the sample

Among the 471 subjects who entered this study, 292 had at least one DSM-IV-TR diagnosis, 151 had somatoform disorders (defined by DSM-IV-TR), and 200 had somatic symptom and related disorders (defined by DSM-5). All had a mean age of 44.68 (SD 13.82) years; mean BMI of 23.25 (SD 3.94) kg/m^2^; and mean duration of psychiatric illness of 2.05 (SD 4.10) years. Moreover, there were 172 male subjects (37.15 %), 187 (39.70 %) who lived in urban areas, and 267 (56.69 %) who were married. The mean PHQ-15, BDI-II, and BAI scores were 7.54 (SD 5.73), 14.54 (SD 12.55), and 13.36 (SD 12.67), respectively.

The DSM-IV-TR category of somatoform disorders (*n* = 151) included undifferentiated somatoform disorder (*n* = 126), pain disorder (*n* = 19), and hypochondriasis (*n* = 16). The main diagnoses other than somatoform disorders were major depressive disorder (*n* = 108), generalized anxiety disorder (*n* = 59), panic disorder (*n* = 56), adjustment disorder (*n* = 21), dysthymic disorder (*n* = 20), and mixed depressive and anxiety disorder (*n* = 17). No patients were comorbid with panic disorder and somatoform disorders (as defined by DSM-IV-TR). The most common comorbidities for somatoform disorders and panic disorder were both major depressive disorder (comorbid with somatoform disorders, *n* = 67; with panic disorder, *n* = 9) and generalized anxiety disorder (comorbid with somatoform disorders, *n* = 37; with panic disorder, *n* = 7).

Among the 200 individuals with somatic symptom and related disorders (DSM-5), most had somatic symptom disorder (*n* = 190), 6 had illness anxiety disorder (with most DSM-IV hypochondriasis subjects diagnosed as having DSM-5 somatic symptom disorder), and 4 had psychological factors affecting their medical conditions.

### Reliability and validity

The results of exploratory factor analysis are shown in Table 1. Four major factors (cardiopulmonary [factor 1], pain-fatigue [factor 2], gastrointestinal [factor 3], dysmenorrhea [factor 4]) were identified. However, factor 4 consisted of only 1 item (item 4), so item 4 was excluded from further analysis (note-it was also excluded from the analyses of Kroenke et al. and others [2, 35]). The loadings of items 11, 10, 7, and 9 on factor 1 were 0.661–0.799; items 3, 2, 6, 8, 14, and 15 on factor 2 were 0.524–0.799; and items 13, 1, 12, and 5 on factor 3 were 0.428–0.715. Additional file 1: Table S1 reveals the results of confirmatory factor analysis. The analysis supports the slightly better performance of our 3-factor model than the original 3-factor model by Kroenke et al. [2]. The test-retest reliability of the PHQ-15 total, factor 1, factor 2, and factor 3 was 0.930, 0.928, 0.803, and 0.911, respectively, and the internal consistency was 0.861, 0.811, 0.781, and 0.637. Among the 3 factors, test-retest reliability of factor 2 was a little lower than that of factor 3 while the internal consistency of factor 3 was a little lower than that of factor 2.

**Table 1:** Exploratory factor analysis of the PHQ-15: factor loading of each item

	Factor 1 (Cardiopulmonary)	Factor 2 (Pain-fatigue)	Factor 3 (Gastrointestinal)	Factor 4 (Dysmenorrhea)
PHQ11: Shortness of breath	0.799			
PHQ10: Palpitation	0.738			
PHQ07: Chest pain	0.709			
PHQ09: Fainting spells	0.661			
PHQ03: Pain in extremities		0.689		
PHQ02: Back pain		0.662		
PHQ06: Headache		0.638		
PHQ08: Dizziness		0.603		
PHQ14: Sleep disturbance		0.594		
PHQ15: Fatigue		0.524		0.520
PHQ13: Nausea or indigestion			0.715	
PHQ01: Stomach pain			0.651	
PHQ12: Constipation or diarrhea			0.612	
PHQ05: Sexual problems			0.428	
PHQ04: Menstrual cramps				0.822

Items with factor loading <0.4 were excluded

*PHQ-15* Patient Health Questionnaire-15

The correlations between PHQ-15 and BDI-II and BAI were moderate to high (Table 2). The correlation coefficient between the PHQ-15 total score and BAI score was 0.721, and between the PHQ-15 total score and BDI-II score was 0.619. The inter-correlations and correlations of the 3 major factors of the PHQ-15 with BDI-II and BAI ranged from 0.4 to 0.7.

**Table 2:** Correlation between the PHQ-15 and the BDI-II, BAI

	Factor 1 (Cardiopulmonary)	Factor 2 (Pain-fatigue)	Factor 3 (Gastrointestinal)	BDI-II	BAI
PHQ-15 total	0.814	0.911	0.777	0.619	0.721
Factor 1 (Cardiopulmonary)		0.614	0.505	0.515	0.647
Factor 2 (Pain-fatigue)			0.589	0.604	0.658
Factor 3 (Gastrointestinal)				0.407	0.536
BDI-II					0.706

All *p* values are < 0.001

*PHQ-15* Patient Health Questionnaire-15, *BDI-II* Beck Depression Inventory-II, *BAI* Beck Anxiety Inventory

### The relation with DSM-IV diagnoses

Based on the DSM-IV-TR, subjects were divided into a somatoform disorders group, panic disorder group (without any somatoform diagnosis), other neurosis group, and no psychiatric diagnosis group. Table 3 compares the PHQ-15 scores in the 4 groups. PHQ-15 scores were a little (though not statistically) higher in the panic disorder group than the somatoform disorders group and significantly higher in the above 2 groups than in the other neurosis group and no psychiatric diagnosis group.

**Table 3:** Comparison of the PHQ-15 in subjects with different DSM-IV/DSM-5 diagnosis

DSM-IV	Somatoform disorders (S, *n* = 151)	Panic disorder (P, *n* = 56)	Other neurotic disorders (O, *n* = 85)	No psychiatric disorder (N, *n* = 179)	Statistics		
	mean (±SD)	mean (±SD)	mean (±SD)	mean (±SD)	*F*	*p* value	Comparison
PHQ-15 total	9.85 (±5.98)	10.96 (±6.18)	7.42 (±4.67)	4.56 (±4.15)	38.318	<0.001	S, P > O > N
DSM-5	With somatic symptom and related disorders (*n* = 200)		Without somatic symptom and related disorders (*n* = 271)		Statistics		
	mean (±SD)		mean (±SD)		*t*	*p* value	
PHQ-15 total	10.04 (±6.03)		5.69 (±4.72)		–8.789	<0.001	

*PHQ-15* Patient Health Questionnaire-15

The result of ROC curve analysis to assess the accuracy of somatoform disorders detection with the PHQ-15 is shown in Table 4 and Additional file 2: Figure S1. The AUC was 0.678 and the optimal cutoff was 6/7 (Youden’s index 0.269). At this cut point, the sensitivity-specificity sum was the highest (1.27).

**Table 4:** Cutoffs for somatoform disorders in the DSM-IV, somatic symptom and related disorders in DSM-5, and somatic symptom disorder with different severity in the DSM-5

	DSM-IV somatoform disorders	DSM-5 somatic symptom and related disorders	DSM-5 SSD, at least mild	DSM-5 SSD, at least moderate	DSM-5 SSD, severe
Cutoff of the PHQ-15 total	6/7	4/5	4/5	6/7	12/13
Sensitivity	0.669	0.845	0.847	0.839	0.571
Specificity	0.600	0.487	0.477	0.384	0.721
Youden’s index	0.269	0.332	0.324	0.223	0.292
AUC	0.678	0.725	0.718	0.597	0.598

*AUC* area under the curve of receiver operating characteristic

### The relation with DSM-5 diagnoses

The comparison of PHQ-15 scores between 2 groups of individuals (1 with and 1 without somatic symptom and related disorders [DSM-5]) is shown in Table 3. The somatic symptom and related disorders group actually had a higher PHQ-15 score (those with the diagnosis: mean = 10.04; those without the diagnosis: mean = 5.69).

The ROC curve of the PHQ-15 item scores for detecting somatic symptom and related disorders is also shown in Table 4 and Additional file 2: Figure S1. The AUC (0.725) was higher than that determined for somatoform disorders (DSM-IV-TR). Similarly the cutoff (4/5) and Youden’s index (0.332) were higher than those determined for the DSM-5 diagnosis.

Because most subjects with somatic symptom and related disorders in our study had SSD (which can be categorized as mild, moderate, or severe according to DSM-5 criteria), ROC curve analysis was performed again to determine the cutoffs for somatic symptom disorder of different severity (AUCs and Youden’s indexes are shown in Table 4). The optimal cutoffs were 4/5, 6/7, and 12/13 for mild, moderate, and severe disease, respectively.

## Discussion

Our study had 4 main findings. First, cardiopulmonary, pain-fatigue, and gastrointestinal are the major factors in the Chinese version of the PHQ-15. Second, the Chinese version of the PHQ-15 shows good reliability, and PHQ-15 score shows moderate correlation with BAI and BDI-II scores. Third, the Chinese version of the PHQ-15 cannot distinguish the DSM-IV-TR diagnosis of somatoform disorders from that of panic disorder. Fourth, the Chinese version of the PHQ-15 (cutoff point of 4/5) can be used to screen for somatic symptom and related disorders (DSM-5). These findings support our hypothesis that the PHQ-15 can be used to evaluate somatic symptom and related disorders (DSM-5) in Taiwan.

Three major factors were identified by exploratory factor analysis. Factor 1 included items 11 (shortness of breath), 10 (palpitation), 7 (chest pain), and 9 (fainting spells), which are features of mainly cardiopulmonary disease and panic attack. Therefore, it was designated the cardiopulmonary factor. Factor 2 consisted of items 3 (pain in extremities), 2 (back pain), 6 (headache), 8 (dizziness), 14 (sleep disturbance), and 15 (fatigue), which deal with pain, neurological symptoms, and fatigue; so it was designated the pain-fatigue factor. Phenomenologically, these symptoms are associated with fibromyalgia and chronic fatigue syndrome. Factor 3 included items 13 (nausea due to indigestion), 1 (stomach ache), 12 (constipation or diarrhea), and 5 (sexual problems). Because of its association with the digestive system and irritable bowel syndrome, factor 3 was designated the gastrointestinal factor. Notably, sexual problems belonged to factor 3, and item 4 (menstrual cramps) was an independent factor. After removing item 4 (which is also removed in Kroenke et al.’s original 3-factor model), the discriminant validity of item 15 increased. Previous studies of the structure of the PHQ-15 revealed the existence of 1 − 5 factors (e.g., 4 factors [cardiopulmonary, gastrointestinal, pain, and neurological] by the Hong Kong study [27] and 1 bifactor [35] [1 general factor and 4 specific symptom factors]). The differences may be attributed to cultural and sample source differences (e.g., if more subjects have panic disorder, the cardiopulmonary factor may be more prominent). Our results also indicate a possible overlap between fibromyalgia and chronic fatigue syndrome in Taiwan. This phenomenon warrants further exploration. In our sample, our 3-factor model shows better model fit than the original 3-factor model by Kroenke et al. (Additional file 1: Table S1) [2]. It might be explained by cross-cultural differences (e.g., in Taiwan, dizziness is highly associated with fatigue and pain as noted in another Chinese study) [36].

In our sample, internal consistency and test-retest reliability of PHQ-15 total scores were good and similar to those found in previous reliability studies [4, 6, 27, 37–39]. The PHQ-15 total score and the 3 main factor scores show moderate to high correlation with BDI-II and BAI scores. Many studies have found that somatization is often comorbid with depression and anxiety [20, 40–42] and may explain why the correlation between the PHQ-15, BDI-II, and BAI is not low. Additionally, among self-administered anxiety questionnaires, the BAI places more emphasis on somatic symptoms, which is compatible with the high correlation between the PHQ-15 and BAI scores. From the above, we consider that the Chinese version of PHQ-15 is both valid and reliable.

From the standpoint of the DSM-IV-TR, PHQ-15 score cannot distinguish somatoform disorders from panic disorder, with the cardiopulmonary factor being identified by factor analysis as the main contributor to panic disorder. However, the PHQ-15 can still be used to discriminate between panic/somatoform disorders and other neurosis. The suitable cutoff for somatoform disorders was 6/7 and differs from 2/3, 4/5, 8/9, and 9/10 reported in other studies [1, 7, 8, 43, 44]. Although our results are compatible with previous findings, the impact of panic disorder on the PHQ-15 should be assessed carefully in the context of the DSM-IV-TR.

PHQ-15 scores differed significantly between the group with and the group without somatic symptom disorder (DSM-5). The AUC of the PHQ-15 for screening somatic symptom and related disorders was higher than that for screening somatoform disorders (0.725 vs 0.678). This may be explained by the emphasis on somatic distress in the DSM-5, which leads to panic disorder and somatic symptom and related disorders becoming more common comorbidities. It differs from the rationale used in differentiating between DSM-IV-TR diagnoses. The reduction in optimal cutoff to 4/5 can also be attributed to the comorbidity of panic disorder and somatic symptom and related disorders. Accordingly, we think that PHQ-15 is more suitably applied in the DSM-5 context. Our results also indicate that the PHQ-15 with cutoffs 4/5, 6/7, and 12/13 can be used to assess somatic symptom disorder severity. The sensitivity was higher than the specificity for the first 2 cutpoints; the specificity was higher for the last cutpoint. However, PHQ-15 is not designed to detect SSD specifically and has no question items about SSD criterion B. Some recently developed questionnaires may be more helpful for exploring SSD criterion B, such as the Somatic Symptom Disorder-B Criteria Scale (SSD-12) [45].

The present study has several limitations. First, a huge proportion in our sample had undifferentiated somatoform disorder in DSM-IV-TR (or somatic symptom disorder in DSM-5). Though somatic symptom disorder is the prototype of this category in DSM-5, whether the Chinese version of the PHQ-15 can be used to assess other disorders with somatic symptoms (such as illness anxiety disorder or conversion) still awaits clarification. Second, we did not directly assess functional disorders not defined by psychiatrists (such as chronic fatigue syndrome, fibromyalgia, and irritable bowel syndrome). Therefore, our inferences from this analysis are partially speculative and should be examined in the future. Third, our study sample consisted of psychiatric patients and healthy individuals in communities. But patients followed only in the clinics of other specialists were not enrolled. Because some patients with functional disorders do not visit psychiatrists, our sample may not be representative of individuals with somatic distress. Inclusion of these patients in future investigations may increase our understanding of the application of the PHQ-15. Fourth, since the cardiopulmonary factor is the main panic disorder-related and anxiety disorder-related subdomain in the PHQ-15, PHQ-15 does not seem to be a specific tool for somatic symptom and related disorders. Finally, we did not examine the inter-rater reliability for the DSM-5-based diagnostic interview.

## Conclusions

Our results support the hypothesis that the Chinese version of PHQ-15 is applicable in Taiwan. The PHQ-15 is more suitable to use in assessing somatic symptom and related disorders (DSM-5) than somatoform disorders (DSM-IV). We believe that this tool will be helpful in screening, evaluating the severity of somatic symptoms, and evaluating the treatment response of patients with somatic symptoms in Taiwan. From a research perspective, we expect to find associations of physiological or psychological indices with the PHQ-15 score that increase our understanding of somatic distress. The relationship between some functional disorders not defined by psychiatrists and the PHQ-15 is also worthy of investigation.

## Additional files

Additional file 1: Table S1. Confirmatory factor analysis and goodness of fit statistics for the different models of PHQ-15. (DOC 32 kb)
Additional file 2: Figure S1. ROC curve: Using PHQ-15 for distinguishing patients in DSM-IV/DSM-5 diagnosis. (DOC 68 kb)
