# Supplementary material for: The relation between the patient health questionnaire-15 and DSM somatic diagnoses
Source: BMC Psychiatry. 2016 Oct 18;16:351. doi: 10.1186/s12888-016-1068-2 (PMC5070166; doi:10.1186/s12888-016-1068-2)
Supplement: Additional file 1: Table S1. — Confirmatory factor analysis and goodness of fit statistics for the different models of PHQ-15. (DOC 32 kb) [file 12888_2016_1068_MOESM1_ESM.doc]

Supplementary Table 1

Confirmatory factor analysis and goodness of fit statistics for the different models of PHQ-15 (N = 471)

| Goodness of fit indexes^1^ | Different factorial model of PHQ-15 | |
| --- | --- | --- |
|  | Our 3-factor model | Kroenke’s 3-factor model |
| d.f | 74 | 62 |
| χ² | 230.25 | 264.09 |
| CFI | 0.925 | 0.900 |
| GFI | 0.935 | 0.924 |
| NNFI | 0.908 | 0.874 |
| RMSEA | 0.067 | 0.083 |
| 90 % CI RMSEA | (0.057, 0.077) | (0.073, 0.094) |
| χ² (d.f.) differences models | Reference | 21.35 (12), p = 0.045 |

Goodness of fit indexes and conventional acceptable criteria: CFI (Comparative Fit Index) > 0.95; GFI (Goodness of Fit Index) > 0.9 ; NNFI (Non-Normed Fit Index) > 0.9; RMSEA (Root Mean Square Error of Approximation) < 0.08

Our 3-factor model contains cardiopulmonary (items 7, 9, 10, and 11), pain-fatigue (items 2, 3, 6, 8, 14, and 15), and gastrointestinal (items 1, 5, 12, and 13) factors

Kroenke’s 3-factor model contains cardiopulmonary (items 7, 8, 9, 10, and 11), pain-fatigue (items 2, 3, 6, 14, and 15), and gastrointestinal (items 1, 12, and 13) factors
