# Supplementary material for: The relation between the patient health questionnaire-15 and DSM somatic diagnoses
Source: BMC Psychiatry. 2016 Oct 18;16:351. doi: 10.1186/s12888-016-1068-2 (PMC5070166; doi:10.1186/s12888-016-1068-2)
Supplement: Additional file 2: Figure S1. — ROC curve: Using PHQ-15 for distinguishing patients in DSM-IV/DSM-5 diagnosis. (DOC 68 kb) [file 12888_2016_1068_MOESM2_ESM.doc]

Supplementary Figure 1

*ROC curve: Using PHQ-15 for distinguishing patients in DSM-IV / DSM-5 diagnosis*

1. (b)


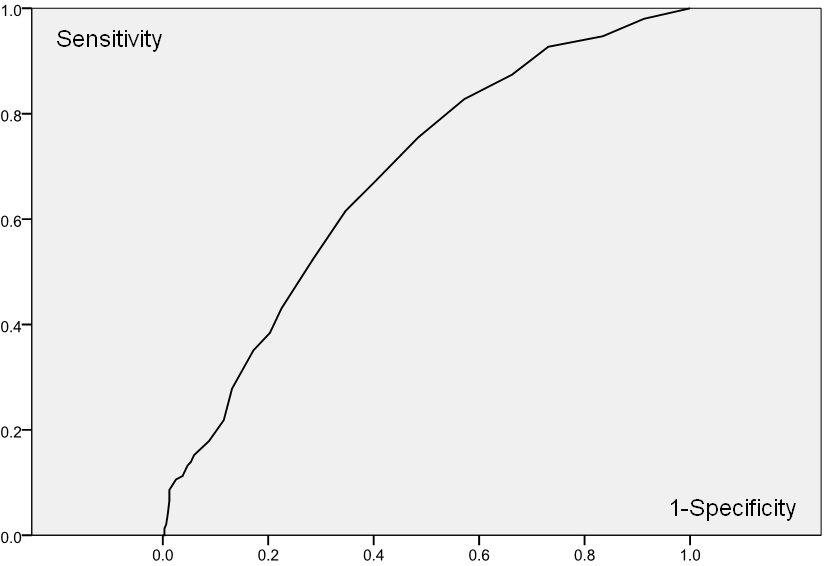

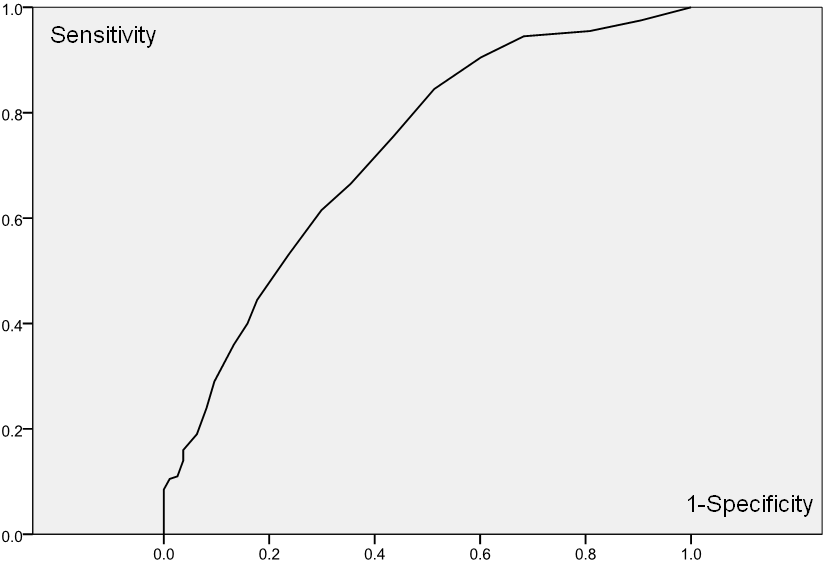


(a) Using PHQ-15 for distinguishing patients with somatoform disorders in DSM-IV

AUC=0.678,Cutoff 6/7, Youden's index = 0.269

(b) Using PHQ-15 for distinguishing patients with somatic symptom and related disorders in DSM-5

AUC=0.725,Cutoff 4/5, Youden's index = 0.332

PHQ-15, Patient Health Questionnaire-15; AUC, area under the curve of receiver operating characteristic
